# 3D Map Combined with Transthoracic Echocardiography for Ablation of Premature Ventricular Contractions/Ventricular Arrhythmia from Papillary Muscle: A Technical Report

**DOI:** 10.3390/jcm13216358

**Published:** 2024-10-24

**Authors:** Gozal Mirzeyeva, Matthias Heukäufer, Sarah Janschel, Daniel Schneppe, Ramin Ebrahimi, Marcus Dörr, Márcio Galindo Kiuchi, Piotr Futyma, Martin Martinek, Helmut Pürerfellner, Christian Templin, Shaojie Chen

**Affiliations:** 1 Department of Internal Medicine B (Cardiology, Angiology, Pneumology and Internal Intensive Care Medicine), University Medicine Greifswald, Ferdinand-Sauerbruch-Straße, 17475 Greifswald, Germany; gozal.mirzayeva@med.uni-greifswald.de (G.M.); matthias.heukaeufer@med.uni-greifswald.de (M.H.); sarah.janschel@med.uni-greifswald.de (S.J.); daniel.schneppe@med.uni-greifswald.de (D.S.); ramin.ebrahimi@med.uni-greifswald.de (R.E.); marcus.doerr@med.uni-greifswald.de (M.D.); christian.templin@med.uni-greifswald.de (C.T.); 2 School of Medicine-Royal Perth Hospital Unit, University of Western Australia, Perth, WA 6000, Australia; marciokiuchi@gmail.com; 3 St. Joseph’s Heart Rhythm Center, Medical College, University of Rzeszów, 35-326 Rzeszow, Poland; piotr.futyma@gmail.com; 4 Department of Internal Medicine 2—Cardiology, Angiology, and Intensive Care, Akademisches Lehrkrankenhaus, Ordensklinikum Linz Elisabethinen, 4020 Linz, Austria; martin.martinek@ordensklinikum.at (M.M.); helmut.puererfellner@ordensklinikum.at (H.P.)

**Keywords:** 3D electroanatomic mapping, transthoracic echocardiography, catheter ablation, premature ventricular contractions, ventricular papillary muscle

## Abstract

Ventricular arrhythmias originating from the papillary muscle of the ventricles are complex clinical problems. Catheter ablation has the potential to cure these arrhythmias. However, the procedure is usually challenging due to the specific anatomy, catheter instability and difficulty in localization of the origin of the arrhythmias. Intracardiac echocardiography (ICE) has been reported to be the suitable imaging method for assessing the location of focus in papillary muscles. We used transthoracic echocardiography (TTE), as a noninvasive cost-effective imaging supporting modality, in combination with 3D mapping to guide the exact localization and successful ablation of papillary muscle-originating premature ventricular contractions (PVCs).

## 1. Introduction

Premature ventricular contractions (PVCs) are a common clinical problem. Papillary muscles (PMs) have been recognized as a potential site of origin of ventricular arrhythmias (VAs)/PVCs with specific electrophysiological features.

Catheter ablation is an effective and therapeutic management option for symptomatic patients with a high PVC/VA burden [1,2,3]. However, the ablation of PM-originating PVCs/VAs has been associated with lower acute and long-term success rates mainly due to the complex anatomy, difficult manipulation and catheter instability, and poor localization of the site of origin [4].

Intracardiac echocardiography (ICE) has been reported to be the suitable imaging method for assessing the location of focus in PMs [4,5,6]. We reported the use of transthoracic echocardiography (TTE) in combination with 3D mapping to guide the exact localization and successful ablation of PM-originating PVCs.

## 2. Methods and Results

A 40-year-old female patient was admitted to our emergency department for recurrent palpitations. Frequent PVCs had been diagnosed for three years. Reversible causes (e.g., ischemic heart disease, electrolyte disturbance, hyperthyroidism, etc.) of PVCs had been ruled out.

**Figure 1:**
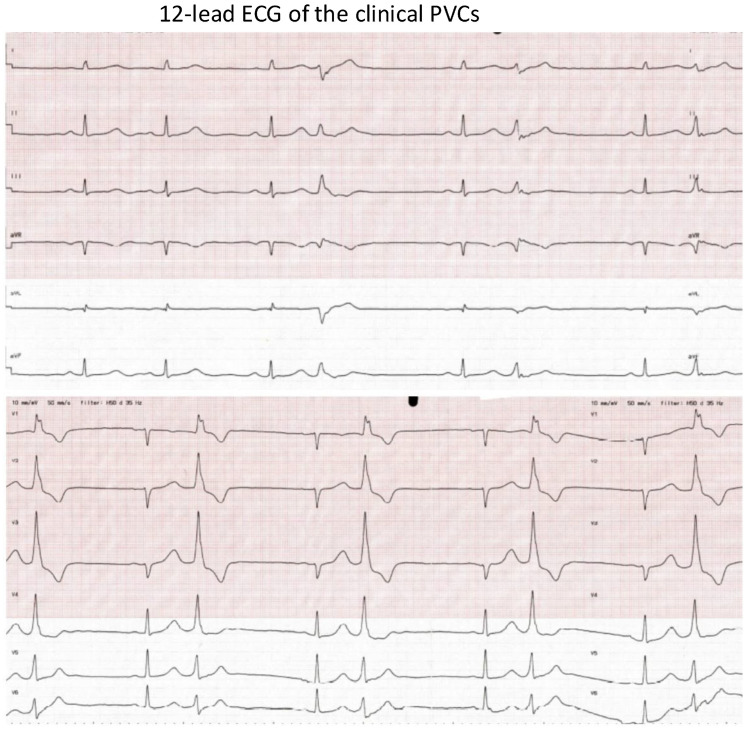
The 12-lead ECG of the clinical PVCs. ECG speed: 50 mm/s. The morphology of the PVCs: (1) RBBB morphology; (2) V1: Rr’; (3) inferior axis; (4) QRS duration: 140 ms. Suspected PM-originating PVCs.

The 12-lead ECG showed sinus rhythms with frequent monomorphic PVCs, sometimes occurring as a bigeminal rhythm (Figure 1). The patient initially had a PVC burden of 9% and this was increased to 15% on 24 h Holter despite antiarrhythmic drug therapy. The spirometry text showed evidence of significantly reduced exercise tolerance and an increase in PVCs during exercise. Echocardiography and a cardiac magnetic resonance scan showed a normal-sized heart, mild mitral insufficiency, and preserved systolic function.

### Mapping and Ablation of the PVCs

After discussion, the decision was made to proceed with catheter ablation. Written informed consent was provided.

**Figure 2:**
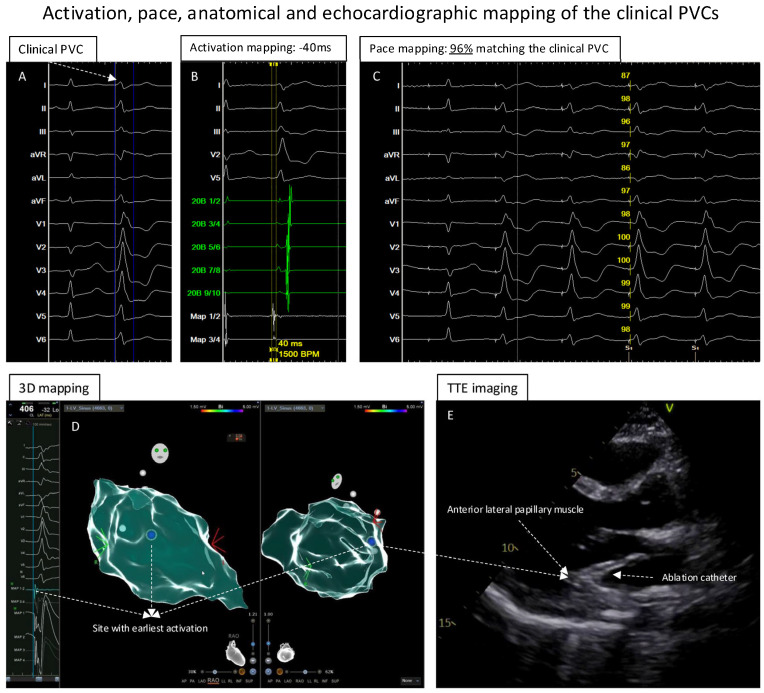
Activation, pace, anatomical and echocardiographic mapping of clinical PVCs. (**A**): The clinical PVC documented in the template. (**B**): The activation mapping of the PVCs showed the earliest local activation with timing of −40 ms before the QRS of the PVCs. (**C**): At the earliest activation site, the pace-mapping technique showed 96% matching score of the clinical PVCs. (**D**): Electroanatomic mapping of the PVCs in LV, the blue point presented the site with earliest activation and excellent unipolar electrograms. (**E**): While mapping the earliest activation site, TTE showed that the catheter was positioned at the anterior–lateral papillary muscle of the LV.

On the day of the procedure, the 12-lead ECG was repeated, and the morphology of the PVCs was identical to the previous ECG documentation (Figure 2A). Based on the ECG, papillary muscle-originating PVC was suspected.

The procedure was performed by the Team of Rhythmology at University Medicine Greifswald. After obtaining femoral vein access, one diagnostic multipolar catheter (6F, Inquiry, Abbott, Chicago, IL, USA) was positioned in the coronary sinus. One transseptal puncture (CARTO VIZIGO Sheath, 8.5F, Biosense Webster; HeartSpan Transseptal Needle, Merit Medical, South Jordan, UT, USA) was performed under fluoroscopic guidance and pressure monitoring. Intravenous heparin was given to maintain the activated clotting time of >300 s during the procedure.

A 3-dimensional (3D) electroanatomic left ventricle (LV) map was created using the multipolar PENTARAY High-Density diagnostic catheter (PENTARAY, CARTO 3 system, Biosense Webster, Irvine, CA, USA) by fast anatomical mapping (FAM). Thereafter, point-by-point acquisition of electrograms using a 3.5 mm irrigated tip catheter (Thermacool SmartTouch SF, Biosense Webster) showed early activation of the PVCs at the area of the anterior–lateral LV.

**Figure 3:**
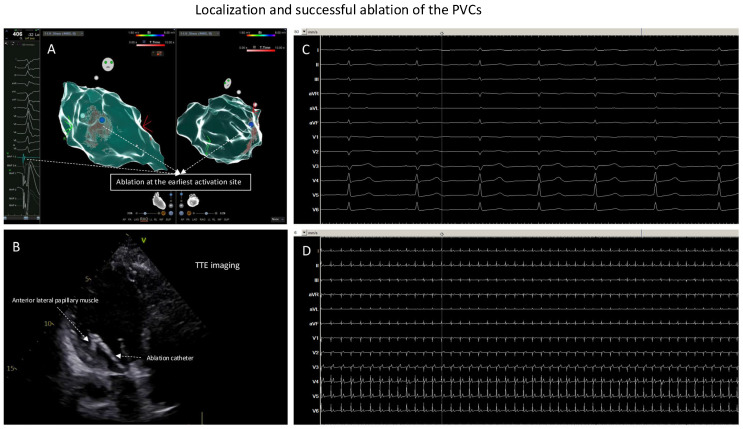
Localization and successful ablation of the PVCs. (**A**): Ablation at the earliest activation site (the blue point). (**B**): At the earliest activation site, TTE monitoring of the ablation catheter was positioned at anterior–lateral papillary muscle of the LV; here, ablation led to elimination of the clinical PVCs. (**C**): ECG documentation, ECG speed 50 mm/s, during or after isoprenaline infusion and with pacing maneuvers: no more inducible PVCs. (**D**): ECG documentation, ECG speed 6 mm/s, during or after isoprenaline infusion and with pacing maneuvers: no more inducible PVCs.

Because of the suspicion of papillary muscle-originating PVCs, TTE was performed to help localize the catheter position. Further point-by-point activation mapping in the region of interest showed the earliest local activation of −40 ms during the clinical PVCs (Figure 2B); the pace mapping at the site with the earliest local activation showed 96% matching with the 12-lead template of the clinical PVCs (Figure 2C). The site with the earliest local activation was revealed to be the tip of the anterior–lateral papillary muscle in the LV under TTE monitoring (Figure 2D,E; videoclips are submitted as Supplementary Materials); here, ablation (Figure 3A,B) with 40 W/60 s per application showed a good response, finally eliminating the clinical PVCs. After the ablation, isoprenaline infusion was administered and programmed stimulation was performed; no PVCs could be induced including the 30 min waiting time (Figure 3B,D).

Twenty-four hours of in-hospital continuous ECG monitoring documented no clinical PVCs. The patient was discharged without any complications. The mid-term follow up showed no recurrent PVCs.

## 3. Discussion

ECG morphologies can help localize the origin of the arrhythmias. Arrhythmias from the left ventricle present right bundle branch block (RBBB) morphology in lead V1. Idiopathic left ventricular arrhythmias arising from the left fascicles, mitral anulus, outflow tract or PMs may have a similar ECG morphology. A narrow QRS (<130 ms) and rR’ pattern in V1 are characteristics of fascicular arrhythmias as they are near the conduction system [7]. In contrast, broad QRS and absence of rR’ are characteristics observed in PM arrhythmias. The VAs from mitral annulus are characterized by positive concordance in V1–V6 leads. Typically, outflow-tract VAs show an inferior axis, and the precordial transition varies depending on the left or right outflow-tract origin [8].

Transthoracic echocardiography (TTE) is a routine part of the diagnostic workflow. Each cardiologist has TTE skills; therefore, TTE use for papillary muscle PVC ablation could be a viable option, and it provides good visualization of the anatomy and function of the heart. Due to the aforementioned difficulties in ablating PM VAs, ICE has been reported to be the suitable imaging method for assessing the location of focus in papillary muscles. However, for interventional electrophysiologists, not everyone is used to ICE manipulation, and economical possibilities in different countries or regions can be another issue. As compared with ICE, TTE may serve as a noninvasive imaging modality to guide the EP ablation procedures without compromising image quality and can be performed not only by electrophysiologist. On the other hand, possible shortcomings of using TTE in the EP lab should also be mentioned; for example, standard TTE projections are not always possible in the EP lab, and the presence of additional people in the EP lab may add complexity to the procedure. A poor acoustic window can also occur when using TTE; in this case, TEE or ICE can be good alternatives.

## 4. Summary

**Figure 4:**
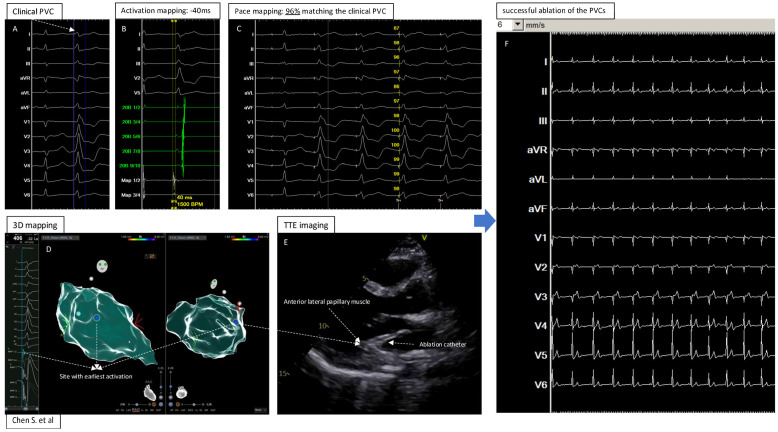
Central illustration: 3D Electroanatomic Mapping in Combination with Transthoracic Echocardiography for Localization and Ablation of Premature Ventricular Contractions from the Left Ventricular Papillary Muscle. (**A**): The clinical PVC documented by 12 lead ECG; (**B**): Activation mapping showed −40ms (earliest) before the QRS of the PVC; (**C**): Pace mapping at the site with earliest activation showed 96% matching the clinical PVC; (**D**): Electroanatomic 3D mapping of the PVC in left ventricle, blue point showed the site with earliest activation and best pacemap of the PVC; (**E**): TTE imaging to visualize the heart and catheter position at the “successful site”; (**F**): Successful ablation and elimination of the clinical PVC.

In summary (Figure 4, central illustration), this report shows the feasibility of using TTE, as a noninvasive cost-effective imaging supporting modality, to describe the exact location of focus and successfully guide the catheter ablation of anterior–lateral papillary muscle-originating PVCs. We acknowledge that this is merely a feasibility report in such a clinical setting, and further studies should be warranted.

## Data Availability

Data are contained within the article and Supplementary Materials.

## Supplementary Materials

The following supporting information can be downloaded at: https://www.mdpi.com/article/10.3390/jcm13216358/s1.
